# Border Line Definition Using Hyperspectral Imaging in Colorectal Resections

**DOI:** 10.3390/cancers14051188

**Published:** 2022-02-25

**Authors:** Boris Jansen-Winkeln, Michelle Dvorak, Hannes Köhler, Marianne Maktabi, Matthias Mehdorn, Claire Chalopin, Michele Diana, Ines Gockel, Manuel Barberio

**Affiliations:** 1 Department of Visceral, Transplant, Thoracic, and Vascular Surgery, University Hospital of Leipzig AöR, 04103 Leipzig, Germany; jacquelineisabellemiche.dvorak@medizin.uni-leipzig.de (M.D.); matthias.mehdorn@medizin.uni-leipzig.de (M.M.); ines.gockel@medizin.uni-leipzig.de (I.G.); 2 Department of General, Visceral and Oncological Surgery, St. Georg Hospital, 04129 Leipzig, Germany; 3 Innovation Center Computer-Assisted Surgery (ICCAS), Leipzig University, 04103 Leipzig, Germany; hannes.koehler@medizin.uni-leipzig.de (H.K.); marianne.maktabi@medizin.uni-leipzig.de (M.M.); claire.chalopin@medizin.uni-leipzig.de (C.C.); 4 Research Institute against Digestive Cancer (IRCAD), 67091 Strasbourg, France; michele.diana@ircad.fr (M.D.); manuel.barberio@ircad.fr (M.B.); 5 ICube Laboratory, Photonics Instrumentation for Health, University of Strasbourg, 67400 Strasbourg, France; 6 Department of General Surgery, Card. G. Panico Hospital, 73039 Tricase, Italy

**Keywords:** hyperspectral imaging (HSI), anastomotic leak (AL), tissue perfusion, tissue oxygenation, intraoperative imaging, colorectal resection

## Abstract

**Simple Summary:**

Good oxygenation of both bowel ends is an important prerequisite to promote anastomotic healing after colorectal resections. Bowel oxygenation is usually assessed clinically. Hyperspectral imaging is a contactless and contrast-free tool that allows quantifying tissue oxygen intraoperatively. In this study, the results of 105 colorectal resections with hyperspectral imaging are reported.

**Abstract:**

Background: A perfusion deficit is a well-defined and intraoperatively influenceable cause of anastomotic leak (AL). Current intraoperative perfusion assessment methods do not provide objective and quantitative results. In this study, the ability of hyperspectral imaging (HSI) to quantify tissue oxygenation intraoperatively was assessed. Methods: 115 patients undergoing colorectal resections were included in the final analysis. Before anastomotic formation, the bowel was extracted and the resection line was outlined and imaged using a compact HSI camera, in order to provide instantaneously quantitative perfusion assessment. Results: In 105 patients, a clear demarcation line was visible with HSI one minute after marginal artery transection, reaching a plateau after 3 min. In 58 (55.2%) patients, the clinically determined transection line matched with HSI. In 23 (21.9%) patients, the clinically established resection margin was entirely within the less perfused area. In 24 patients (22.8%), the HSI transection line had an irregular course and crossed the clinically established resection line. In four cases, HSI disclosed a clinically undetected lesion of the marginal artery. Conclusions: Intraoperative HSI is safe, well reproducible, and does not disrupt the surgical workflow. It also quantifies bowel surface perfusion. HSI might become an intraoperative guidance tool, potentially preventing postoperative complications.

## 1. Introduction

Colorectal cancer (CRC) represents the most common gastrointestinal malignancy worldwide, with 1.8 million new cases per year [1]. Currently, anastomotic leakage (AL) following colorectal resections has an incidence ranging from 2 to 19% [2], influencing morbidity and mortality negatively, and worsening global oncological outcomes [3,4]. AL has a multifactorial etiology with a number of well-defined patient risk factors, such as morbid obesity, tobacco use, male sex, immunosuppression, and emergency surgery [5]. However, poor blood perfusion at future anastomotic margins represents a recognized cause for impaired anastomotic healing. Indeed, intraoperative anastomotic perfusion assessment based on clinical factors exclusively (e.g., serosa color, bowel peristalsis, etc.) is unreliable [6]. To improve the operator’s perception, several new technologies have been recently introduced into clinical practice in order to overcome the human vision’s limits [7].

Among those, fluorescence angiography (FA) with indocyanine green (ICG) as a fluorophore has been extensively used to assess bowel viability during colorectal resections [8,9,10], and some recent evidence has shown promising results in terms of AL rate reduction [11,12,13,14,15]. However, FA does not provide universally accepted quantitative results. Consequently, its interpretation is currently mainly subjective. Alternatively, hyperspectral imaging (HSI) can deliver a real-time snapshot of living tissue in a contrast-free manner. It can also detect and quantify blood flow with a higher precision degree than FA [16,17,18].

Recently, it was proven that HSI could quantify bowel perfusion at the proximal resection margin during colorectal resections [19], and it has delivered results comparable to FA in a small patient cohort [20]. In contrast to FA, HSI does not need any dye and it is repeatable without interferences.

In the current study, extending the same clinical protocol described in our previous work [19] to a larger patient series, HSI was used to measure bowel perfusion at the proximal resection site in order to compare it to clinical assessment during colorectal resections at a specialized colorectal unit of a German University Hospital.

## 2. Materials and Methods

### 2.1. Study Population

In this prospective, non-randomized single-arm study, all patients undergoing colorectal resections at the University Hospital of Leipzig from February 2018 to May 2020 were included. Pregnancy, age less than 18 years, inability to give written informed consent, emergency surgery, peritoneal carcinomatosis, recurrent malignancy, or indication for cytoreductive surgery were the exclusion criteria for this study. The study received approval by the local ethical committee of the University of Leipzig (026/18-ek) and was registered at Clinicaltrials.gov (NCT04226781).

Clinical, histopathological, and hyperspectral data were analyzed. In particular, AL was diagnosed either radiologically using a CT-scan, which was ordered if AL clinical suspicion was raised (such as increased infection blood parameters, clinical signs for sepsis, and purulent/fecal discharge from drains), or endoscopically in patients with rectal resections (all patients after low anterior resection received an endoscopic control on postoperative day 6–8 according to our clinical guidelines).

### 2.2. Hyperspectral Imaging System

Hyperspectral data were acquired using the commercially available TIVITA^®^ Tissue system (Diaspective Vision GmbH, Am Salzhaff, Germany), which analyzes data within a 500 to 1000 nm spectral range and a 640 by 480-pixel spatial range. Data acquisition occurs in a virtually real-time fashion (acquisition time <10 s), and the integrated software provides a set of color-coded images as the immediate output, representing physiological tissue parameters intraoperatively (i.e., tissue oxygenation (StO_2_ in %), near-infrared perfusion index (NIR), organ hemoglobin index (OHI), and tissue water index (TWI)). The latter three are represented in arbitrary units from 0 to 100, and the algorithms to calculate these parameters have been thoroughly explained before [21,22]. To prevent any external light contamination, environmental lights were turned off during HSI data acquisition.

### 2.3. Surgical Technique and Data Acquisition

Regardless of the surgical approach, all resections were performed in a standardized fashion. A medial–to–lateral bowel dissection was performed, and, for oncological resections, colorectal oncological paradigms, such as central ligation of the district vessels, complete mesocolic excision, or (partial or total) mesorectal excisions, were followed. In left-sided resections, a mini-laparotomy was performed, and the colon was extracted through a wound retractor. At this point, the marginal artery was divided, and the future proximal transection margin was chosen and marked by the operating surgeon, based on clinical judgment exclusively. In right colonic resections, since we performed extracorporeal anastomoses, the procedure was similar in terms of the choice and imaging of the resection margin. In detail, an HSI image of the colon with a ruler next to it was taken before cutting the marginal artery (Min 0). The mesocolon was then completely dissected. The “cold steel test” (arterial bleeding from the marginal artery) was performed (timer started). If the “cold steel test” was successful, the surgeon marked the clinically determined transection line by positioning the tip of scissors in its proximity. HSI images were acquired each minute for a total of 5 min (Min 1—one minute after cutting the marginal artery—to Min 5—five minutes after). Following this, the bowel was resected according to the demarcation line between the adequately and the poorly perfused bowel. The anastomoses were created successively as follows: in case of right colectomies, a hand-sewn end–to–end ileotransversostomy; for left hemicolectomies, a stapled end–to–end descendorectostomy; and, for rectal resections, a stapled side–to–end descendorectostomy.

### 2.4. Data Processing

Based on hyperspectral StO_2_ false-color images, the borderline between the adequately and the poorly perfused intestinal surface was determined. The borderline was defined as a 50% decrease in the measured values between the maximum and minimum values, but at least 80% StO_2_.

**Figure 1:**
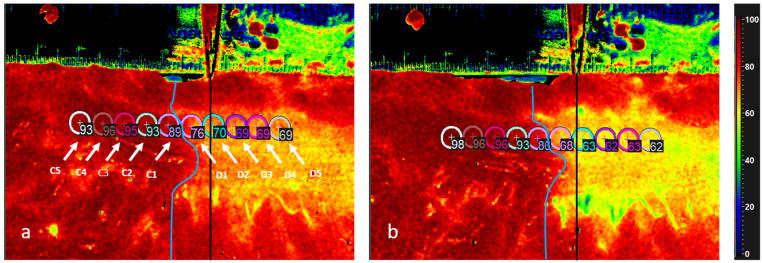
HSI image for StO_2_ of the colon with scale bar on the top. The black line (indicated by the scissors) marks the transection line placed by the surgeon, and the blue line illustrates the course of the real border zone. Values of all markers (5 centrally and 5 distally of the border zone) 1 min (**a**) and 3 min after devascularization (**b**). D5 is the most distal one and C5 is the most central one. Values distal to the border zone decline while the proximal values increase. The distance between the marked transection line by the surgeon and the real borderline is clearly shown.

Using the camera analysis software, 10 regions of interest (ROI) (each 5 mm in diameter) were placed along the bowel—5 ROIs centrally (named C_1_ to C_5_) and 5 ROIs distally of the transition line (named D_1_ to D_5_). The software calculates the average of StO_2_ values within each ROI automatically. This parameter corresponds to superficial tissue oxygenation (Figure 1). The same analysis was performed using the false-color images of the NIR index, which corresponds to the oxygenation of the deeper tissue (up to 5 mm in depth).

The distance (in mm) between the clinically determined transection line, previously marked with the tip of the scissors, and the borderline defined via HSI was measured on the ruler. At the end, we characterized the course of the borderline by measuring the distance between the most proximal and distal points, where the StO_2_ value was still over 80%. This cut-off value was set subjectively. As a benchmark, the healthy colon was measured. Values of 85 to 95% were measured in previous works [19]. In the patients included in this study, StO_2_ in the healthy colon before resection was 90.8% on average (SD 4.470). As a result, under the premise that the best perfusion is just good enough, we selected 80% in our study. These values were compared to the subjective cut-off value of 75% StO_2_.

### 2.5. Statistical Analysis

HSI and patient data were transferred to Microsoft Excel Version 16.0 (Microsoft Corporation, Washington, DC, USA) and statistically evaluated with IBM SPSS Statistics Standard v24 (IBM Corporation, Chicago, IL, USA). The program was used to test for normal distribution (Kolmogorov-Smirnov-test), and conduct parametric ANOVA tests and *t*-tests where appropriate. Statistical significance was reached for *p* values < 0.01.

## 3. Results

### Perioperative Data Analysis

**Figure 2:**
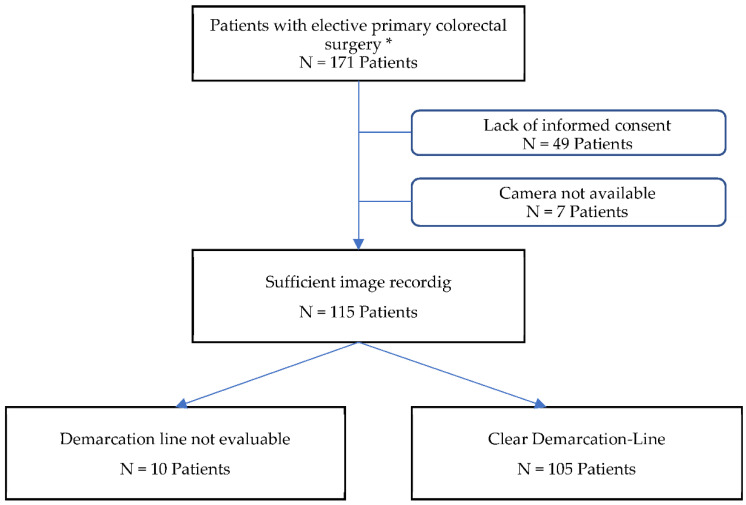
Flow–chart of patient selection. * Exclusion criteria: Pregnancy, age less than 18 years, inability to give written informed consent, emergency surgery, peritoneal carcinomatosis, recurrent malignancy, or indication for cytoreductive surgery.

There were 171 patients eligible for study inclusion (Figure 2). The cohort consisted of 111 (65%) men and 60 (35%) women. Patients exhibited a median age of 62 years (range: 21–86). One hundred and twenty-two (71.3%) patients underwent surgery due to malignancy and 49 (28.7%) for diverticular disease. Seventy-four patients (43.3%) underwent anterior rectal resections, and 67 (39.2%) left and 30 (17.5) right hemicolectomies. A total of 121 (70.8%) procedures were performed laparoscopically, and 38 (22.2%) robotically; six (3.5%) were conversions to open surgery, and six (3.5%) were primary open surgical procedures. A total of 56 patients were excluded (i.e., 49 patients due to lack of informed consent and 7 patients because the HSI camera was unavailable). Finally, 115 patients were considered for the definitive analysis. Data from 115 patients (74 men/41 women) with a mean age of 63 years (range: 27–86) and a BMI of 26 kg/m^2^ (range: 16–41) were analyzed (Table 1). Eighty-seven percent of the patients had an American Society of Anesthesiologists (ASA) grade II score. Surgeries were divided into left hemicolectomy (including sigmoid resection) (n = 49), rectal resection (n = 54)—48 with protective ileostomy, and right hemicolectomy (n = 12). Among all diagnoses, 71 were malignancies. Most procedures were performed laparoscopically (n = 72) or robotically (n = 38). Among these, there were 2 conversions (i.e., one due to intraoperative decision–making in favor of multivisceral resection and abscess, and one due to massive adhesions caused by previous open abdominal surgery). The median operating time was 225 min (range: 100–737). The longest surgery (i.e., 737 min) was a robotic rectal resection in a patient with a BMI of 40 kg/m^2^. The case was converted to open surgery and ultimately received a transanal total mesorectal excision (TaTME) with a hand-sewn coloanal anastomosis (CAA). In 10 cases, the results of the HSI measurement could not be included in the further calculation. In four cases, a clear assessment of the intestinal wall was not possible due to pronounced epiploic appendices. Consequently, the intestinal serosa was not sufficiently exposed in order to place the necessary markers. In six other cases, no clear demarcation line was visible, neither at the beginning nor at the end of the measurement. Although there was a perfusion gradient, a close examination of the specimen revealed a small vascular bridge in the region of an appendix, which ensured a low residual perfusion. All operations were performed without relevant intraoperative complications, and the patients were stable with regards to the anesthesiology cardiopulmonary parameters during the procedure. During the postoperative course, two patients developed Clavien–Dindo [23] grade 3 complications and had to be operated on again. One of them received a gastrectomy due to an acute upper gastrointestinal bleeding for a gastric carcinoma found incidentally, and one received an ileostomy for a small anastomotic leak after a left hemicolectomy. One patient (Clavien–Dindo grade 4) developed a pulmonary embolism postoperatively (Table 2).

A total of eight patients developed AL (6.9%) (Table 3). Overall, all patients with AL displayed a longer in-hospital stay (20–41 days), except for the patient who received percutaneous drainage alone. This patient remained hospitalized for 9 days.

Cardiovascular parameters were recorded during the HSI measurements. The blood pressure was stable in the normal range in all patients (median systolic 120 mmHg (range: 100–140), diastolic 70 mmHg (range: 40–90)). The administration of catecholamines during measurements was necessary in 52 (49.5%) patients, and then continued at low doses (median 0.029 µg/kg/min (range: 0.001–0.173)). The peripherally measured median oxygen saturation (SaO_2_) was 98% (range: 92–99) with a concomitant median inspiratory oxygen concentration of 44% (range: 27–61).

**Figure 3:**
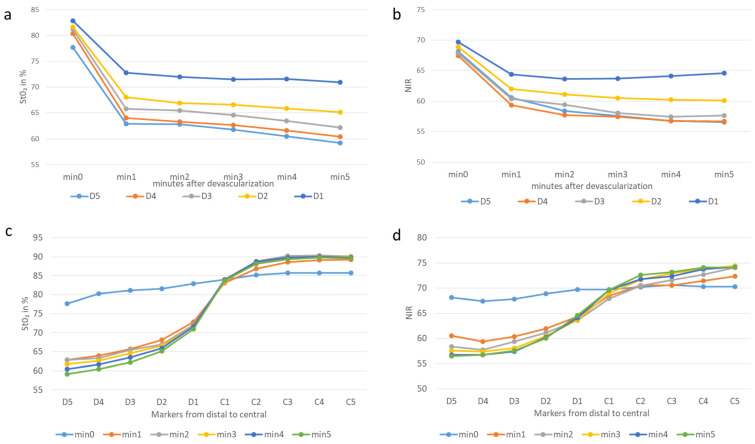
Graphic illustration of the changes in tissue oxygen saturation and near-infrared perfusion at the different measurement points over time (**a**) StO_2_, (**b**) NIR and along the longitudinal axis of the colon 3 min after devascularization, (**c**) StO_2_, (**d**) NIR. All markers had a diameter of 5 mm and illustrate the value of tissue perfusion. The demarcation line is located between C1 and D1; D5 is the most distal marker and C5 the most central one. a and b illustrate the biggest significant drops distal to the border zone 1 min after devascularization. Illustrations c and d demonstrate the course between the markers and time.

The mean cumulative StO_2_ at Min 0 (before cutting the marginal artery) was 83.3% (both centrally and distally), and it was significantly higher than that at Min 1 (77.2%; *p* < 0.0001). Here, StO_2_ dropped by an average of 19.2% (SD 7.73) at points D2–5, which were significant overall distal points at the time of one minute (*p* < 0.0001). It continued until minute three, with a significant drop of 20.8% (SD 8.3687) (measuring points D2–D5). Consequently, the demarcation line was already visible after one minute and became even clearer up to minute three, after which no significant change occurred (Figure 3).

In the central region, on the other hand, an opposite effect was visible. Here, StO_2_ increased after the marginal arcade had been cut. On average, StO_2_ increased by 4.4% (SD 1.86). This was statistically significant (*p* < 0.0001) at measurement points C2–5. At measurement point C1, the value remained relatively constant over time (Figure 3).

StO_2_ in patients with AL was not statistically different at either one or three minutes after the transection of the marginal artery, with mean values of 86.5% (SD ± 5.68) vs. 85.1% (SD ± 7.47) in the groups with AL and without AL, respectively. At minute three, patients with AL had higher TWI (median 66.9; in contrast with 62.6 in the group without AL) and StO_2_ (median 90.4; others 88.0). However, NIR was lower (median 66.9; others 70.6). Patients with the longest duration of surgery exhibited lower StO_2_ (median 87.6; others 88.2), NIR (68.0; others 70.1), and TWI (median 62.1; others 63.0). These measurements had no statistical significance. There was also no statistical correlation between StO_2_ and BMI or StO_2_ and the duration of surgery.

**Figure 4:**
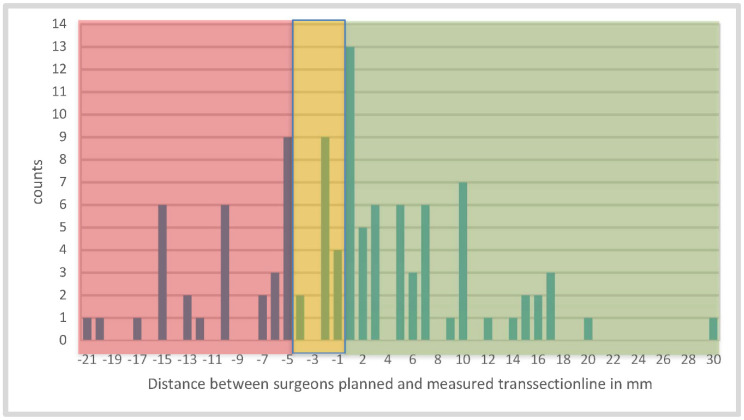
Illustration of the distance between the transection lines planned and measured by the surgeon in mm 3 min after devascularization; (**green**) demonstrates good perfusion and (**red**) indicates poor perfusion. We decided to expand the tolerance area (**yellow**) to minus 4 mm, because of the diameter of the tip of the scissors, which is 4 mm and can cause incorrect measurements. Finally, in 73 cases, the surgeon placed the top of the scissors in the correct or acceptable perfused area.

**Figure 5:**
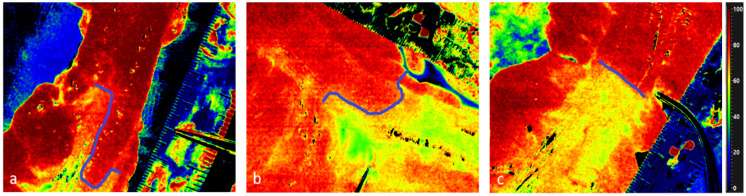
Illustration of the border zone with StO_2_ in HSI. The scissor on the scale bar indicates surgeons planed resection line. The blue line helps to visualize the course of the border zone. (**a**) and (**b**) clearly show that the border zone can be wider and runs out. (**c**) is nearly straight.

Additionally, the border zone detected via HSI was compared to the planned surgical resection line (highlighted by the tip of the instrument/scissors). It was noticeable that this zone did not always run straight in a linear fashion, but often extended over a wide area. In some cases, the border zone extended up to 25 mm (median 4 mm (range: 1–25)) in width. For this reason, we compared the most central and distal points of the HSI transition zone with the position of the instrument as visually determined by the surgeon. The surgical line was set more distally to the entire border zone in 23 patients (21.9%) and within the well-perfused area in 58 patients (55.2%). The tip of the scissors, which was used as a measuring landmark, was 4 mm in diameter, and only distances of >4 mm were included in the evaluation (Figure 4). In 24 patients (22.8%), the surgical dissection line was placed in the area between the proximal and distal border zone points (because of the non-straight course), and, consequently, in a partially poorly perfused area (Figure 5). In contrast, comparing the surgical resection line to the border zone at 75% StO_2_, 76 (72.4%) of the measurement results were in the well-perfused area, and 29 (27.6%) were in the less-perfused region. Selecting again the range less than or equal to 4 mm and above, 13 (12.4%) assessments were 5 mm or more in the tissue with oxygen saturation below 75%.

## 4. Discussion

In the current investigation, we were able to assess and quantify tissue oxygenation and perfusion during colorectal resections in a large patient series. In fact, after the dissection of the marginal artery, the well-perfused areas were clearly distinguishable from the less perfused ones only after one minute. In line with our previous observations made in a smaller patient series [19], perfusion decreased progressively within the first 3 min, reaching its lowest value at that time point, and subsequently remained constant until minute 5.

Poor blood flow at the future anastomotic site represents a well-known modifiable AL cause [24]. ALs represent a dreaded complication following colorectal surgery, with a tremendous impact on patient lives, negatively affecting global oncological outcomes [25].

Detecting and quantifying the extent of ischemia relying on clinical parameters exclusively provides inaccurate estimations [6]. For this reason, several methods to more objectively measure bowel blood flow intraoperatively have been proposed in the past [26,27]. Among these methods, FA is certainly the one with the highest reproducibility and with the most promising results [9]. However, the method still suffers from consistent subjective interpretation biases and depends on intravenous ICG injection. On the other hand, HSI provides quantified results without the necessity for injecting a contrast agent. Other compromising factors for anastomotic healing, such as tension on the anastomosis, were not taken into account. In addition, it is a methodological problem that the final anastomosis was not assessed with HSI, only the proximal end. The final anastomosis was technically not visible in the majority of surgeries (rectal resection) with the camera system used. This is a problem in all studies investigating such anastomoses [11,14,19,28,29].

Furthermore, the use of hyperspectral imaging during colorectal surgical procedures unveiled interesting pathophysiological phenomena regarding capillary blood flow redistribution following marginal artery transection. Our current results clearly demonstrated an increase in the perfusion of the central zones after the division of the mesocolon at the distal transection point.

Remarkably, in four patients undergoing anterior resection, the entire bowel section was poorly perfused (StO_2_ < 50%) at the time of initial imaging, and the color of the colon subjectively showed no obvious ischemia. Additionally, the “cold steel test” yielded a weakly positive result. For this reason, the colon was further mobilized, and his measurement was performed further centrally, where it showed acceptable values. This allowed the surgeon to modify the resection margin and to carry on the surgical procedure. Given the existing high variability within the inferior mesenteric artery district, in particular concerning the course and presence of the marginal artery of Drummond or the arc of Riolan [30,31], we hypothesized that, in these four patients, both vascular structures were either absent or inadvertently damaged during the procedure, or impaired due to the presence of an atherosclerotic disease. Nonetheless, in these particular cases, the intraoperative use of HSI represented an added value and allowed performing the anastomosis in a well-perfused area, possibly preventing a potential AL. The shape of the demarcation zone is remarkable, with a partly substantial width. A circular assessment of the bowel would be optimal here. However, the observation of the semi-circumference already underlines the importance of placing the resection above the proximal end of the demarcation line.

Ten of the 115 patients included in our study had to be excluded because of inconclusive HSI analysis. In six patients, no clear demarcation line was visible with HSI, because perfusion appeared to be evenly distributed. On closer examination, this was due to an incomplete dissection of the mesocolon or collateral perfusion obtained via an epiploic appendix. On the contrary, in four patients, the HSI assessment was insufficient, because the mesenteric and epiploic fat covered large portions of the serosa, impairing light tissue interaction. This represents a common limitation of all optical imaging techniques, including near-infrared fluorescence angiography, since they can exclusively analyze the superficial portion of the tissue. The subjective interpretation of FA results indeed represents a consistent limitation of this method. For this reason, quantitative FA analysis (slope or time-to-peak of the fluorescent signal) was introduced [32,33]. Nonetheless, to date, the current FA devices cannot provide a quantitative assessment of perfusion, and this must be performed as postprocessing analysis. Remarkably, in a previous experimental study, quantitative FA was compared to HSI using robust ischemia biomarkers as “ground truth”. Within this controlled experimental setting, HSI showed significantly higher accuracy than FA [17].

Interestingly, we noticed that the resection line based on clinical judgment exclusively was placed correctly in half of the patients (55.2%) only. These observations confirm the inadequacy of the human sensorium in assessing the ischemia degree of the gastrointestinal tract and the need for intraoperative technological aids to overcome this limitation [6]. It is also possible that our cut-off value of 80% was set too high. This oxygen saturation is indeed exceeded by healthy bowels, with a mean of 90.8% StO_2_. However, it is not known which threshold still allows for safe anastomotic healing. When comparing to the limit value of 75%, it is noticeable that 12% of the surgically determined resection lines are 5 mm or more in the area with a lower StO_2_. On the other hand, it can perhaps be deduced that the naked eye of the surgeon is unable to distinguish perfusion between 75 and 80%. Better oxygen saturation may be a favorable condition for good anastomotic healing. However, the thresholds are unknown and need to be established by means of big data in the future. Another factor to discuss is the Hawthorne effect. The surgeon must handle the HSI camera during the procedure and is able to see the HSI measurement results directly on the screen. Methodical blinding is technically difficult to achieve within a clinical trial. As a result, an influence bias on data assessment should be taken into account [34].

The AL rate encountered in our series is in line with the incidence found in the literature and all patients suffering from anastomotic complications presented at least one of the intrinsic risk factors stated in the introduction. However, given the set-up of our study, in which the resection line was always moved to the optimally perfused zone, following HSI assessment, the real impact of this technology on reducing the AL rate remains unclear and the AL we observed can potentially be due to other risk factors, rather than suboptimal perfusion.

It should be noted that, in our study, the planned transection line was placed mostly within the well perfused areas detected with HSI (73 cases, see Figure 4). In case this was set within the poorly perfused areas (42 patients), the maximum distance from the planned transection line was, at most, 21 mm. Considering that, in the majority of the cases, a circular stapler was used to fashion the anastomose, at least 1 cm of the poorly perfused bowel would have been resected, falling within the proximal “doughnut”. For this reason, it is unclear the impact that such a minimal discrepancy could have had on the AL rate.

## 5. Conclusions

The current study confirms the feasibility of quantifying bowel perfusion using his during colorectal surgery in a large series, and it makes this technology a candidate as a valid alternative to FA and a respectable future intraoperative imaging tool. However, the current lack of real-time video imaging and of a commercially available laparoscopic HSI camera represent real limitations for the evolution of this technology on a large scale. Nevertheless, we are sure that near-future technological improvements will allow the surgical community to overcome such constraints.

Finally, the real value of intraoperative perfusion assessment using HSI to reduce complications remains unclear, and larger trials with a randomized set-up are necessary to answer this crucial question.

## Figures and Tables

**Table 1:** Preoperative findings, N = 115.

Variables	N (%)
ASA classification	
Grade I	7 (6.1)
Grade II	87 (75.7)
Grade III	21 (18.2)
Comorbidities	
Renal failure	10 (8.7)
Cardiovascular disease	80 (69.6)
Metabolic disease	20 (17.4)
Alcohol/Nicotine abuse	22 (19.1)
Pulmonary diseases	7 (6.1)
Others	80 (69.6)
None	11 (9.6)
Previous abdominal surgeries	50 (43.5)
Neoadjuvant therapy	25 (21.7)
Medication	
Antihypertensive drugs	80 (69.6)
Antiplatelet drugs	61 (53.1)
Antidiabetics	17 (14.8)
Others	74 (64.3)
No medication	7 (6.1)

**Table 2:** Postoperative findings, N = 115.

Variables	N (%)	Median
In-hospital stay (days)	-	10 (6–41)
Histopathological entity		
malignant	71 (61.7%)	
benign	44 (38.3%)	
UICC classification		
Stage 0 (after neoadjuvant therapy)	8 (7.0%)	
Stage I	20 (17.4%)	
Stage II	24 (20.9%)	
Stage III	16 (13.9%)	
Stage IV	3 (2.6%)	
Clavien-Dindo classification (CDC)		
Grade IIIa	5 (4.3%)	
Grade IIIb	2 (1.7%)	
Grade IV	1 (0.9%)	
Anastomotic leakage	8 (6.9%)	

**Table 3:** Patient characteristics of patients with anastomotic leaks.

	Procedure	Therapy	ASA	Neoadjuvant Therapy	Mean StO_2_ after 3 min	Mean NIR after 3 min	Other Risk Factors
Pat. 1	LH	DI	2	-	83	69	Nicotine abuse
Pat. 2	LH	PCD	3	-	87	76	Diabetes, cardiovascular disease
Pat. 3	H	PCD	2	CRT	85	41	Cardiovascular disease
Pat. 4	RR and DI	EVAC	2	-	88	57	-
Pat. 5	RR and DI	EVAC	2	-	87	68	Cardiovascular disease, BMI > 40 kg/m^2^
Pat. 6	RR and DI	EVAC	2	-	80	69	Cardiovascular disease, BMI > 40 kg/m^2^
Pat. 7	RR and DI	EVAC	2	CRT	89	63	-
Pat. 8	RR and DI	PCD	3	CRT	91	80	Nicotine abuse

StO_2_—Tissue oxygenation; NIR—Near-infrared perfusion; LH—Left hemicolectomy; DI—Diverting ileostomy; PCD—Percutaneous drainage; EVAC—Endo-vacuum therapy; CRT—Chemoradiotherapy.

## Data Availability

The data presented in this study are available on request from the corresponding author.
